# Editorial Notes

**Published:** 1935

**Authors:** 


### Editorial Notes

**Dr. T. S. Hele, Master of Emmanuel College, Cambridge.**

The election of Dr. T. S. Hele to be Master of Emmanuel College, Cambridge, is the first occasion on which a former resident officer at a Bristol hospital has become head of a college in Oxford or Cambridge.

Dr. Thomas Shirley Hele is University Lecturer in Bio-chemistry. After a distinguished career at Cambridge, where he took a first-class in both parts of the Natural Sciences Tripos, and a period of study at St. Bartholomew's Hospital he came to the Bristol Royal Infirmary as house surgeon, 1910–11.

His subsequent career has been an academic one as a University lecturer in Cambridge and as an important officer, Bursar, of his college. Dr. Hele served with the R.A.M.C. in Salonika during the war.

His many Bristol Royal Infirmary friends will wish him all success in his new appointment, and will no doubt visit him as opportunity arises in the pomp and splendour of the Master's Lodge at Emmanuel.

**International Congress of the History of Medicine, Madrid, 1935.**

The Tenth International Congress of the History of Medicine was held in Madrid from the 22nd to the 30th September, 1935, under the Presidency of Professor Gregorio Marañón. Nearly 500 members representing 42 nations attended the Congress. The principal communications dealt with

Arab Medicine in Spain, Arab Medicine in general, and Medicine in Eastern countries, Medicine in America during its discovery and colonization, and Folk Medicine.

An important section of the work of the Congress was an exhibition of manuscripts, documents, instruments, books and other objects bearing on the subjects under discussion. The exhibit of The Wellcome Historical Medical Museum included some interesting dioramas, namely Nicolas Monardes of Seville (*circa* 1512–1588) depicted working in his private Museum of Curiosities on his celebrated book upon *Medicinal Plants of South America*—completed in 1574; a historic incident connected with the discovery of cinchona bark as a remedy for malaria, which took place at the Vice-regal Palace at Lima (*circa* 1630); a Hispano-Moresque apothecary's shop; and an interior view of the hospital of Santa Cruz at Toledo as it appeared in the sixteenth century.

Apart from the scientific sessions there were many interesting excursions and entertainments, notably to Toledo, the Escorial and to Alcalá de Henares. The last named is the old University town in which Cervantes was born, and the famous Polyglot or Complutensian Bible was printed. The University has now been incorporated with that of Madrid. On the occasion of the visit to Alcalá de Henares a special Academic Assembly was held in the Paraninfo or Great Hall of the University, where Honorary degrees were conferred on several distinguished foreign members of the Congress. Sir Humphry Rolleston was thus honoured amongst the British representatives.

There were receptions at the National Palace by the President of the Republic, President Zamora, and by the Mayor and Council of Madrid at the Town

Hall. The beautiful climate and elevated situation of Madrid (2,800 feet) made the visit exceptionally delightful. The hotel accommodation was very good, and the food everywhere most appetizing. The traditional courtesy of the Spanish was appreciated in full measure. The most impressive part of the visit, however, was the splendid rebuilding which is going on in Madrid. The width and lighting of the streets, both by day and night, exceed anything that has been contemplated by town planners in this country. The modern buildings, although plain, are singularly beautiful. Perhaps the building which awakened the greatest feelings of envy was a newly-built infants' school. The new University City is planned on a vast scale, which scarcely seemed justifiable until one realized that there are already 3,000 students of medicine alone in Madrid. One surprise was encountered, namely, that within thirty miles of Madrid five months of winter sports may be enjoyed amid the most comfortable surroundings in the Sierra de Guadarrama.

**Dr. G. Parker, Honorary Member of the Society.**

AT a meeting held on Wednesday, 13th November, Dr. George Parker was elected an Honorary Member of the Society in recognition of his great services as Hon. Medical Librarian in the University Library. Dr. Parker has been a member of the Bristol Medico-Chirurgical Society since 1887 and Medical Librarian since October, 1930, when he accepted the office on the resignation of Dr. W. A. Smith. He was President in 1915–16, and last year the University marked its appreciation of his services to the University as Lecturer in Forensic Medicine for many years and as Medical Librarian by conferring on him the degree of M.D. *Honoris Causâ.*

**British Medical Association Meeting, Melbourne, 1935.**

AT the Annual Meeting held in Melbourne Bristol was well represented by Professor and Mrs. Hey Groves and Professor and Mrs. Parry. It is particularly gratifying that Bristol surgery should have been distinguished by the Honorary Fellowship of the Royal Australasian College of Surgeons, which was conferred upon Professor Hey Groves. He was President of the section of Orthopædics, and had the honour of delivering the first Hamilton Russell Memorial Oration on "The Romance of Surgery." He also delivered a Popular Lecture, "The Valley of Dry Bones," and read a paper on "The Treatment of Fractures of the Neck of the Femur." Professor Parry occupied the important position of Secretary to the section of Public Health and Hygiene. We are looking forward to hearing an account of their travels (illustrated by a film) at the meeting of the Bath, Bristol and Somerset Branch of the British Medical Association on Wednesday, 29th January, 1936.

